# Genetic and pathogenic difference between Streptococcus agalactiae serotype Ia fish and human isolates

**DOI:** 10.1186/s12866-016-0794-4

**Published:** 2016-08-02

**Authors:** Chishih Chu, Pei-Yu Huang, Hung-Ming Chen, Ying-Hsiang Wang, I-An Tsai, Chih-Cheng Lu, Che-Chun Chen

**Affiliations:** 1 Department of Microbiology, Immunology, and Biopharmaceutics, National Chiayi University, Chiayi, 60004 Taiwan, ROC; 2 Department of Aquatic Biosciences, National Chiayi University, Chiayi, 60004 Taiwan, ROC; 3 Department of Pediatrics, Chang Gung Memorial Hospital, Chiayi, Taiwan, ROC

**Keywords:** Streptococcosis, *Streptococcus agalactiae* (GBS), Pulsotype, MLST, Serotype, Tilapia

## Abstract

**Background:**

*Streptococcus agalactiae* (GBS) is a common pathogen to infect newborn, woman, the elderly, and immuno-compromised human and fish. 37 fish isolates and 554 human isolates of the GBS in 2007–2012 were investigated in serotypes, antibiotic susceptibility, genetic difference and pathogenicity to tilapia.

**Results:**

PCR serotyping determined serotype Ia for all fish GBS isolates and only in 3.2 % (3–4.2 %) human isolates. For fish isolates, all consisted a plasmid less than 6 kb and belonged to ST7 type, which includes mainly pulsotypes I and Ia, with a difference in a deletion at the largest DNA fragment. These fish isolates were susceptible to all antimicrobials tested in 2007 and increased in non-susceptibility to penicillin, and resistance to clindamycin and ceftriaxone in 2011. Differing in pulsotype and lacking plasmid from fish isolates, human serotype Ia isolates were separated into eight pulsotypes II–IX. Main clone ST23 included pulsotypes II and IIa (50 %) and ST483 consisted of pulsotype III. Human serotype Ia isolates were all susceptible to ceftriaxone and penicillin and few were resistant to erythromycin, azithromycin, clindamycin, levofloxacin and moxifloxacine with the resistant rate of 20 % or less. Using tilapia to analyze the pathogenesis, fish isolates could cause more severe symptoms, including hemorrhage of the pectoral fin, hemorrhage of the gill, and viscous black and common scites, and mortality (>95 % for pulsotype I) than the human isolates (<30 %); however, the fish pulostype Ia isolate 912 with deletion caused less symptoms and the lowest mortality (<50 %) than pulsotype I isolates.

**Conclusion:**

Genetic, pathogenic, and antimicrobial differences demonstrate diverse origin of human and fish serotype Ia isolates. The pulsotype Ia of fish serotype Ia isolates may be used as vaccine strains to prevent the GBS infection in fish.

**Electronic supplementary material:**

The online version of this article (doi:10.1186/s12866-016-0794-4) contains supplementary material, which is available to authorized users.

## Background

*Streptococcus* are pathogenic to cause streptococcal disease for humans and animals. Among *Streptococcus* spp., Gram-positive *Streptococcus agalactiae* (group B *streptococcus*; GBS) is a normal human gastrointestinal and genitourinary flora. Therefore, GBS infect more commonly the vaginas of women, especially more prevalent in the pregnant woman than those of non-pregnant woman [1] and causes early-onset or late-onset sepsis and meningitis in newborns. In fish, the major pathogenic species that cause streptococcosis are *S. agalactiae* (GBS), *S. dysgalactiae*, *S. iniaee* and *Lactococcus garvieae*, which also infect human. Multiplex PCR has been developed to differentiate these species and to identify serotypes [2], especially the GBS serotypes [3, 4].

Streptococcosis is an important disease in fish. After infection, fishes may suffer meningitis and septicemia in common [5]; however, other syndromes may be associated with fish species [6]. Streptococcal infection has been reported in rainbow trout in 1957 [7] and later on in various fishes, including *O. aura* × *O. nilotica* hybrid fish [8], *Mugil cephalus* L. [9], *Anguilla japonica*, *Seriola quinqueradiata* [10], *Paralichthys olivaceus* [11], *Ictalurus punctatus* [12], hybrid-striped bass (*Morone chrysops* × *Morone saxatilis*) [13], *Sebastes schlegeli* [6], *Seriola dumerili* and *S. lalandi* [14]. Using antibiotics to treat streptococcal infection in fish, resistance rate to erythromycin, clarithromycin, and azithromycin was less than 15 % for fish isolates [15]. Recently, human GBS has gradually become resistant to clindamycin and erythromycin and differed in resistance rate among countries and sources [16, 17].

In GBS, pathogenicity to fish may be associated with serotypes. For examples, serotypes serotype Ia is more pathogenic than serotype III [18], serotype Ia and Ib more prevalent in seafood [19], serotype Ib in Queensland grouper and serotype II in wild fish and stingrays in Australia [20]. Genetic and plasmid variations may change the host virulence and specificity. As diverse genetic sources, plasmids have been found to carry genes for drug resistance and virulence in various streptococcal species [21–23]. Furthermore, GBS virulence to fish also depends on environmental conditions, such as temperature above 26 °C increases the GBS virulence to tilapia [24]. Recent study reported that an increase in temperature from 28 to 35 °C cause near two-fold mortality in tilapia and regulate the gene expression, such as up-regulation of the proinflammatory genes for cyclooxygenase-2, Il-1β and TNF-α [25]. Recently, we reported the prevalence change in serotypes and mutations in GyrA and ParC causing fluoroquinolone resistance of GBS human isolates [26, 27]. Genomic analysis of human and fish isolates suggest transfer of GBS between human and fish [28].

To investigate the possible zoonotic infection of GBS, serotypic, genomic and pathogenicity differences between human and fish GBS isolates collected from the diseased fishes in aquaculture farms and patients from nearby hospital.

## Methods

### Bacterial isolates and biochemical identification

Bacteria were isolated from diseased fishes, including mullet (*Mugil cephalus*), tilapia (*Oreochromis hybrids*), big-scale liza (*Liza macrolepis*), bass [*Lates calcarifer* (giant seaperch), *Bidyanus bidyanus* (silver perch), *Lateolabrax japonicus* (Japanese seaperch), *Morone saxatilis* (striped bass) and *Scortum barcoo* (Jade perch)] and other species [*Thunnus albacares* (yellowfin), *Acanthopagrus schlegelii* (blackhead seabream) and *Epinephelus lanceolatus* (brindle grouper)] in Lutsao, Dongshih, Yizhu, Budai and Puzih of Chiayi county and Kouhu of Yulin county, and Tainan County in 2007–2012. Bacterial species were identified by Gram-staining, catalase testing, the Rapid ID 32 STREP system (Bio-Mérieux Inc, France), and PCR amplification. 554 human isolates from 2007 to 2012 were identified in Chiayi Chang Gung Memorial Hospital (CGMH) located at Puzih of Chiayi County near the center of the fish farms. This study was approved by the research ethics committee of CGMH (97-0077B and 99-3958B). The protocols for fish experiment were performed according to the guidelines of the Animal Use Protocol and the Institutional Animal Care and Use Committee (Protocol 97017) of the National Chiayi University.

### PCR identification of bacterial species and serotypes of GBS

Single colony was taken into Brian Heart Infusion (BHI) broth and total DNA was purified from overnight bacterial cultures using the Genomic DNA purification kit (Quality Systems Inc., Taiwan). Primers for bacterial identification are listed in Additional file 1: Table S1 and were designed by the combination and modification of previous primers [29]. The 25-μl PCR reaction mixture contained 1X PCR buffer, 0.2 mM dNTPs, 1.5 mM MgCl_2_, 0.2 μM primers, and 0.5 U Taq DNA polymerase. The PCR conditions were as follows: predenaturation at 94 °C for 2 min; 25 cycles of denaturation at 94 °C for 30 s, annealing at 55 °C for 45 s, and extension at 72 °C for 45 s; and a final extension at 72 °C for 5 min. Serotyping of the GBS isolates was performed according to methods described previously [4]. PCR products were separated by 0.5 % TBE and 1.5 % agarose at 50 V for 1.5 h. After staining with ethidium bromide, gel images were taken under ultraviolent light illumination.

### Antimicrobial susceptibility

Antibiotics that are commonly used in treatment of fish and human infection included amoxicillin, azithromycin, ceftriaxone, clindamycin, doxycycline, erythromycin, florfenicol, levofloxacin, moxifloxacin, oxytetracycline, and tetracycline. After adjusting the bacterial concentration to a 0.5 McFarland standard, the susceptibilities to these antibiotics were determined by the disc method (BD BBL^TM^ Sensi-Disc^TM^; BD Diagnostics, Franklin Lakes, NJ, USA) and the guideline of CLSI standard [30]. Susceptibility to penicillin was measured by the disc method (BD BBL^TM^ Sensi-Disc^TM^; BD Diagnostics, Franklin Lakes, NJ, USA) and the Etest® (BioMérieux, Marcy‑l’Etoile, France). *Streptococcus pneumonia* ATCC49619 was used as the reference strain.

### Genetic analysis of fish and human GBS serotype Ia isolates

The plasmid number was determined by the method of Kado and Liu [31] with a minor modification of the lysis buffer to 0.2 N NaOH and 1.5 % SDS and plasmid size was estimated using 6.6- and 50-kb plasmids of *S.* Choleraesuis strain OU7085. An 600-bp *Sau*3A plasmid DNA fragment from fish isolate 886 was cloned and sequenced. The genotype of each isolate was determined by PFGE analysis. Briefly, overnight bacteria were first embedded in 0.8 % agarose. The plugs were treated with lysozyme and then 1 mg/ml proteinase K at 50 °C. After washing with TE buffer, the plugs were digested with the restriction endonuclease *Sma*I. The macro-DNA fragments were separated by CHEF DRIII (BioRad, Taiwan) using a switching time of 4 s/70 s, 120°, and 6 V for 18 h for the first step and then a switching time of 4 s/70 s, 120°, and 4 V for 6 h. Strains with a banding pattern difference of more than three bands were designated different genotypes, and strains with at least one band difference were designated different subgenotypes [32]. MLST analysis of fish isolates were performed according to the methods described earlier [33] and sequence types (ST) were determined by the *S. agalactiae* MLST database (http://pubmlst.org/sagalactiae/).

### *The virulence of human and fish* GBS *isolates to tilapia*

Seven fish isolates (886, 900, 912, 948, 953–1, 954, and 1004) and seven human isolates (G91, G108, G110, and G116 of serotype Ia as well as G1, G78, and G102 of serotype III) were used to investigate the differences in pathogenicity to tilapia between these human and fish isolates. Six tilapia fishes with the size of 7–9 cm were randomly grouped. Each fish was intraperitoneally injected with approximately 1 × 10^8^ cfu bacteria, and a PBS injection was used as a control. The death number of each group was recorded for two weeks. In experiment I, we investigated the mortality rate and symptoms of human and fish isolates each group were recorded. In experiment II, we only determined the mortality for the isolates at14 days after inoculation.

## Results and discussion

Broad-host-range GBS can infect humans, fish and other animals [34–36]. In Taiwan, Yulin and Chiayi counties are the major aquatic culture regions with bass, mullet and tilapia as the primary fish, in which *L. garvieae* and GBS are main pathogens. In this study, we analyzed genetic and pathogenic differences of the GBS isolates from a hospital and nearby cultured fish farms to investigate the possible zoonotic transmission of GBS between fish and human.

### Serotype distribution of S. agalactiae fish and human isolates

**Fig. 1:**
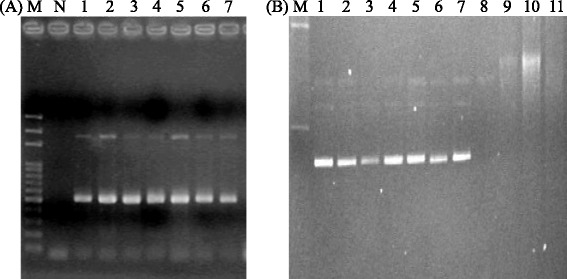
Gel electrophoresis of multiplex PCR products for serotyping (**a**) and plasmids (**b**). A. M: 100 bp size marker, N: negative control, Line 1 to 7 are seven fish isolates 886, 900, 912, 948, 953–1, 954, and 1004. Amplicon sizes of serotype Ia are 1826 and 521 bp. B. M: 6.6 and 50 kb plasmids of *Salmonella* Choleraesuis isolate 7085. Lanes 1 to 7 are fish isolates 886, 948, 1004, 900, 912, 954, and 953–1. lane 8–11 are human isolates G91, G108, G110, and G115

All 37 *S. agalactiae* isolates were mainly isolated in 2011 and belonged to serotype Ia identified by Multiplex PCR (Fig. 1 and Table 1). These isolates were isolated from various important cultured fishes, mainly from tilapia (40.5 %), followed by bass (37.8 %), big scale liza (5.4 %), mullet (8.1 %), and other species (8.1 %) (Table 2). Among 554 human GBS isolates, these were only 20 serotype Ia isolates (3.6 %) with the prevalence of 3.8 % (7/185) in 2007, 3.0 % (5/169) in 2008, 3.7 % (3/81) in 2011 and 4.2 % (5/119) in 2012 (Table 1). Serotype Ia isolates were collected mainly from urine (65 %, 13/20), followed by vagina and genital tract swab, and prefer to infecting female than male (13 vs. 7) (Table 3), suggesting that serotype Ia isolates were not the major serotype to infect human and less invasive. Next, we investigated the genetic relatedness and clonal dissemination of these serotype Ia isolates.

**Table 1:** Prevalence of human and fish *Streptococcus agalactia* serotype Ia isolates and their pulsotypes in four years

Year	2007	2008	2011	2012	Total
Total fish isolates	7		23	7	37
Serotype Ia [N (%)]	7 (100)		23 (100)	7 (100)	37 (100)
Pulsotypes [N (%)]					
I	6 (85.7)		19 (82.6)	5 (71.4)	30 (81.1)
Ia	1 (14.3)		4 (17.4)	0 (0)	5 (13.5)
ND	0 (0)		0 (0)	2 (28.6)	2 (5.4)
Total human isolates	185	169	81	119	554
Serotype Ia [N (%)]	7 (3.8)	5 (3.0)	3 (3.7)	5 (4.2)	20 (3.2)
Pulsotypes [N (%)]					
II	2 (28.6)	2 (40)	1 (33.3)	2 (40)	7 (35)
IIa	2 (28.6)	2 (40)	1 (33.3)		5 (25)
IIb	1 (14.3)				1 (5)
III	1 (14.3)				1 (5)
IV	1 (14.3)				1 (5)
V		1 (20)			1 (5)
VI			1 (33.3)		1 (5)
VII				1 (20)	1 (5)
VIII				1 (20)	1 (5)
IX				1 (20)	1 (5)

**Table 2:** Characterization of *Streptococcus agalactiae* fish isolates

Fish species	Place	2007	2011	2012	Number of each genotype			Number of resistant isolates		
					I	Ia	ND	Penicillin	Ceftriazone	Clindamycin
Mullet	Chiayi	1		1	1/0/0^a^		0/0/1			0/0/0
	Tainan		1		0/1/0			0/1/0		
Tilapia	Chiayi	2	8	3	2/7/3	0/1/0		0/4/0		
	Yunlin	2			2/0/0					
Big scale liza	Chiayi		1	1	0/1/0		0/0/1	0/0/1		0/0/1
Bass	Chiayi	1	9		0/7/0	1/2/0		0/3/0	0/1/0	
	Yunlin		3	1	0/2/1	0/1/0				
Other species	Chiayi	1		1	1/0/1					
	Yunlin		1		0/1/0					
Total		7	23	7	6/19/5	1/4/0	0/0/2	0/8/1	0/1/0	0/0/1

^a^1/0/0 means one isolates in 2007, and 0 in 2011 and 2012

**Table 3:** Characteristics of 20 *Streptococcus agalactiae* Ia human isolates

Strain	Year	Sex	Source^a^	Pulsotype	Resistance to^b^				
					Erythromycin	Azithromycin	Clindamycin	Levofloxacin	Moxifloxacin
G15	2007	M	B	II	-	-	-	+	+
G91	2007	F	U	IIa	-	-	-	-	-
G108	2007	M	U	IIa	-	-	-	-	-
G110	2007	F	U	III	+	+	+	-	-
G116	2007	M	U	II	-	-	-	-	-
G127	2007	M	U	IV	-	-	-	-	-
G176	2007	M	U	IIb	-	-	-	-	-
G233	2008	F	U	II	-	-	-	+	+
G268	2008	M	U	V	-	-	-	-	-
G335	2008	F	U	IIa	-	-	-	-	-
G340	2008	F	U	IIa	-	-	-	-	-
G349	2008	M	U	V	-	-	-	-	-
G645	2011	F	OTH	II	-	-	-	-	-
G649	2011	F	OTH	VI	+	+	+	-	-
G656	2011	F	GTS	IIa	-	-	-	-	-
G684	2012	F	GTS	IX	-	-	-	-	-
G741	2012	F	U	II	-	-	-	-	-
G764	2012	F	U	II	-	-	-	-	-
G772	2012	F	VA	VII	+	+	+	-	-
G800	2012	F	VA	VIII	-	-	+	-	-
Total resistant number [n (%)]					3 (15)	3 (15)	4 (20)	2 (10)	2 (10)

^a^ *B* blood, *U* urine, *GTS* genital tract swab, *VA* vagina, *OTH* others

^b^+: resistance, −: susceptible

### Genotyping and MLST analysis of GBS human isolates

**Fig. 2:**
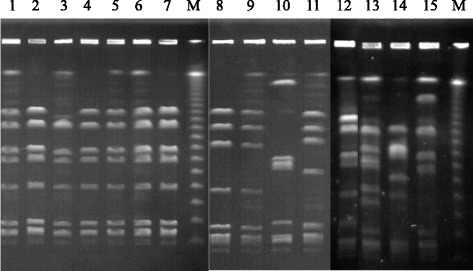
Pulsed-field gel electrophoresis of *Sma*I-digested macro-framents of *S. agalactiae* isolates. M: λ-DNA marker. Line 1–7 are fish isolates 886, 900, 912, 943, 954, 953–1, and 1004. Line 8–15 are human isolates G91, G108, G110, and G116 of serotype Ia, G1 of serotype III, G12 of serotype V, G13 of serotype VI, and G14 of serotype Ib

Phylogenetic relations between serotype Ia human and fish isolates were investigated by three methods: PFGE, plasmid and MLST analysis. PFGE analysis of *Sma*I-digested macro-fragments determined that all fish isolates belonged to pulsotypes I (81.1 %), Ia (13.5 %), and non-typable (5.4 %) and size difference between pulsotype I and Ia was observed in the largest DNA fragment, possibly a 200-kb difference (Fig. 2, Table 2). None of fish pulsotypes was identified in 20 human isolates, which were separated into nine pulsotypes II-IX with two major clones: pulsotypes II (5 isolates) and IIa (5 isolates) (Table 3). These results demonstrate clonal dissemination in fish and human isolates, which differed genetically. All fish isolates contained a plasmid smaller than 6 kb, which was not observed in human isolates tested (Fig. 1, Table 4). Sequence analysis of a plasmid fragment identified a gene encoding plasmid recombination enzyme found in GBS (accession number YP_001586274 and EFV96312) and on 4.1-kb plasmid pER13 of *Streptococcus thermophilus.*

**Table 4:** Characterization of human and fish isolates for fish cytotoxitity test

Source	Strain	Serotype	MLST^a^	Pulsotype	Plasmid	Resistance^b^ to		
						Erythromycin	Azithromycin	Clindamycin
Fish	886	Ia	ST7	I	+	-	-	-
	900		ST7	I	+	-	-	-
	912		ST7	Ia	+	-	-	-
	948		ST7	I	+	-	-	-
	953-1		ST7	I	+	-	-	-
	954		ST7	I	+	-	-	-
	1004		ST7	I	+	-	-	-
Human	G91	Ia	ST23	II	-	-	-	-
	G108		ST23	II	-	-	-	-
	G110		ST483	III	-	+	+	+
	G116		ST23	IIa	-	-	-	-
	G1	III	ND	Ib	-	-	-	-
	G78		ND	IV	-	+	+	+
	G102		ND	V	-	+	+	+

^a^ND: Non-determined. All isolates were collected in 2007

^b^+: resistance, −: susceptible

MLST analysis of the major pulsotypes of fish and human serotype Ia isolates identified that ST types differed between fish and human isolates: ST7 for all fish isolates, ST23 for human pulsotypes II and IIa and ST483 for pulsotype III (Table 3). ST7 is major pathogen to cause disease in tilapia in Asia and has been reported in isolates from tilapia and human [37]. Genetic differences in ST type, pulsotype and plasmid imply that human and fish isolates may vary in pathogenicity to tilapia.

### Antibiotic susceptibility of human and fish serotype Ia isolates

Multidrug resistant isolates have emerged due to the intensive culture of fishes and antimicrobial misuse to control bacterial infection. The antimicrobial resistance patterns of *Streptococcus* spp. differed among mammals, reptiles, amphibians and aquatic animals [15]. The antimicrobial resistance of *Streptococcus* isolates ranged from greater than 85 % resistance to spiramycin, enrofloxacin, and clarithromycin to less than 60 % resistance to erythromycin, azithromycin and amoxicillin. In this study, all fish isolates were susceptible to amoxicillin, doxycycline, oxytetracycline, florfenicol, levofloxacin, and moxifloxacin. Previously, it was reported that the disk diffusion methods using penicillin G disks could not determine penicillin resistant GBS isolates [38]. Indeed, MIC (mg/L) for penicillin tested against 34.8 % (8/23) isolates with penicillin resistance determined by disk method ranged from 0.16 to 0.23, 0.47, and 0.64 in 2011 compared to 0.25 mg/L of *S. pneumonia* ATCC49619 and only one isolate was resistance to ceftriaxone, clindamycin, and erythromycin. An increase of resistance to erythromycin of macrolide, clindamycin of lincosamide, and ceftriaxone and non-susceptible to penicillin that are commonly used in human may be needed to concern.

In compared to other serotypes, serotype Ia isolates were less resistance to antimicrobials tested and emerged resistance to levofloxacin and moxifloxacin [26]. The human GBS Ia isolates were sensitive to penicillin and ceftriaxone while human isolates were higher in resistance to azithromycin, clindamycin, and erythromycin than fish isolates (Tables 2 and 4). Simultaneous mutations in the quinolone resistance-determining regions of *gyrA* and *parC* were observed in two levofloxacin and moxifloxacin resistant isolates [27], which may be same clone from different patients with identical genotypes and antibiogram.

### Pathogenicity analysis of S. agalactiae to tilapia

In tilapia, GBS infection frequently causes meningoencephalitis with high mortality [39]. Such infection occurs while tilapia is over 20 g in weight and grows in the condition of broodstock on-growing and market fish [40]. Pathogenicity analysis to tilapia was performed using 14 fish and human isolates, which characteristics are listed in Table 4. Briefly, all seven fish isolates of pulsotypes I and Ia were almost identical genetically and sensitive to azithromycin, erythromycin, and clindamycin while seven human serotypes Ia and III differed in ST types, pulsotypes, and resistance to azithromycin, erythromycin, and clindamycin.

The symptom appearance in fish is strain- and serotype-dependent. It has been reported that GBS serotype Ia isolates are more pathogenic to tilapia than serotype III isolates [18]. After infecting by GBS fish isolates, tilapia showed three primary symptoms that were viscous black and common ascites (25–91.7 %) being the most prevalent, hemorrhage of the gill (8.3–58.3 %) and hemorrhage of the pectoral fin (8.3–50 %) (Table 5). Considering symptom prevalence in tilapia for fish isolates, pulsotype I isolate 900 was the most virulent, and pulsotype Ia isolate 912 was the least virulent. In human isolates, viscous black and common ascites, hemorrhage of the gill and hemorrhage of the pectoral fin were occasionally observed, for examples, only viscous black and common ascites in one fish infected by serotype Ia isolates G91, G108, and G110 and hemorrhage of the gill and hemorrhage of the pectoral fin infected by serotype G1.

**Table 5:** Death and symptom number of tilapia response to seven serotype Ia fish isolates and seven serotype Ia and III human isolates

Bacterial Source	Serotype	Strain	Repeat^a^	Symptom									Mortality				
				Hemorrhage of pectoral fin			Hemorrhage of gill			Viscous black and common ascites			I^a^			II^b^	
				No.	Mean		No.	Mean		No.	Mean (%)		No.	Mean		Bacterial dose (1 × 10^8^ cfu)	Mean (%)
					No.	%		No.	%					No.	%		
Fish isolates	Control		1	0/6	0/6	0	0/6	0/6	0	0/6	0/6	0	0/6	0/6	0	0	16.7 ± 0
			2	0/6			0/6			0/6			0/6				
	Ia	886	1	0/6	0.5/6	8.3	2/6	2.5/6	41.7	5/6	4/6	66.7	6/6	6/6	100	3.1–3.8	100 ± 0
			2	1/6			3/6			3/6			6/6				
		900	1	2/6	3/6	50	3/6	3.5/6	58.3	5/6	5.5/6	91.7	6/6	6/6	100	2.5–2.6	100 ± 0
			2	4/6			4/6			6/6			6/6				
		912	1	0/6	0.5/6	8.3	0/6	0.5/6	8.3	2/6	1.5/6	25	5/6	3.5/6	58.3	2.1–3.1	54.2 ± 25.0
			2	1/6			1/6			1/6			2/6				
		948	1	3/6	2.5/6	41.7	3/6	2.5/6	41.7	4/6	4/6	66.7	6/6	5.5/6	91.7	3.1–3.8	91.7 ± 16.7
			2	2/6			2/6			4/6			5/6				
		953-1	1	0/6	1/6	16.7	2/6	1.5/6	25	2/6	1.5/6	25	6/6	6/6	100	2.5–2.6	100 ± 0
			2	2/6			1/6			1/6			6/6				
		954	1	0/6	1/6	16.7	3/6	3/6	50	6/6	4.5/6	75	6/6	5/6	83.3	2.1–3.1	54.2 ± 25.0
			2	2/6			3/6			3/6			4/6				
		1004	1	1/6	0.5/6	8.3	1/6	1/6	16.7	5/6	4/6	0	6/6	5/6	83.3	2.1–3.4	95.8 ± 8.3
			2	0/6			1/6			3/6		0	4/6				
Human Isolate	Control		1	0/6	0/6	0	0/6	0/6	0	0/6	0/6	0	1/6	1/6	16.7	0	16.7 ± 0
			2	0/6			0/6			0/6			1/6				
	Ia	G91	1	0/6	0/6	0	0/6	0/6	0	1/6	0.5/6	8.3	1/6	0.5/6	8.3	2.3–2.9	8.3 ± 9.62
			2	0/6			0/6			0/6			0/6				
		G108	1	0/6	0/6	0	0/6	0/6	0	1/6	0.5/6	8.3	0/6	0.5/6	8.3	1.8–2.2	16.7 ± 13.6
			2	0/6			0/6			0/6			1/6				
		G110	1	0/6	0/6	0	0/6	0/6	0	0/6	0.5/6	8.3	1/6	1/6	16.7	2.1–3.0	8.3 ± 8.34
			2	0/6			0/6			1/6			1/6				
	III	G116	1	0/6	0/6	0	0/6	0/6	0	0/6	0/6	0	2/6	1/6	16.7	1.9–2.6	29.2 ± 21.0
			2	0/6			0/6			0/6			0/6				
		G1	1	1/6	0.5/6	8.3	1/6	0.5/6	8.3	0/6	0/6	0	1/6	0.5/6	16.7	1.3–2.0	16.7 ± 13.6
			2	0/6			0/6			0/6			0				
		G78	1	0/6	0/6	0	0/6	0/6	0	0/6	0/6	0	0	0/6	16.7	1.4–1.9	12.5 ± 16.0
			2	0/6			0/6			0/6			1				
		G102	1	0	0/6	0	0/6	0/6	0	0/6	0/6	0	0	0.5/6	16.7	1.3–2.1	12.5 ± 16.0
			2	0			0/6			0/6			0				

^a^6 fish were used in each repeat

^b^Six fish were used for each isolate and each data were average of four repeats

Early reports demonstrated that fish and human isolates caused fish mortality differently. In contrast to the contention that human isolates are more lethal than fish isolates (LD_50_ = 10^6^ CFU/per fish for human isolates vs. LD_50_ = 6.1 × 10^7^ ~ 1.94 × 10^8^ cfu/per fish for fish isolates) [41], fish isolates were more lethal to tilapia than cattle or human isolates at low LD_50_ [24, 37]. With a bacterial dose of 2.1 ~ 3.8 × 10^8^ cfu for fish isolates and 1.3 ~ 3.0 × 10^8^ cfu for human isolates in this study, the mortality rate of tilapia was, on average, below 50 % for fish isolate 912 and over 95 % for the remaining fish isolates in contrast to less than 30 % for all human isolates (Table 5), demonstrating that fish isolates were more virulent to tilapia than human isolates. GBS can infect diverse fish species and cause economic loss in fish farming. Therefore, vaccine is needed to prevent GBS infection. Recently, a vaccine strain with a truncated surface immunogenic protein (tSip) has been constructed against GBS infection [42]. In this study, the fish pulsotype Ia isolate 912 can be a vaccine candidate for tilapia due to low mortality.

The differences in symptom and mortality rate between human and fish isolates are possibly due to growth temperature and genetic differences. Recently, genome analysis of GBS determined more than 15 possible virulence genes homologous to genes pathogenic to human [43]. As virulence factor, β-hemolysin of GBS serotype Ia isolate is involved in the survival in the human macrophage THP-1 cell and enhance the tumor necrosis factor-α release [44]. Furthermore, host factors may also play important role in the defense of pathogen infection. In tilapia, hepcidin (TH) 1–5 can increase resistance to bacteria pathogens through modulation of related cytokines [45] and an increase of T-cell receptor expression of tilapia plays a role in response to GBS infection [46].

## Conclusions

GBS isolates were the pathogens to bass, mullet, and tilapia and increased the resistance to antimicrobials used in human. ST types, pulsotypes and pathogenesis of GBS serotype Ia isolates differed between human and fish isolates, implying impossible transmission between human and fish in this study. The genes on the deletion fragment and plasmid of serotype Ia fish isolates may be responsible for the GBS virulence to tilapia.

## Abbreviations

CGMH, Chiayi Chang Gung Memorial Hospital; CLSI, Clinical and Laboratory Standards Institute; GBS, group B Streptococcus; MLST, multilocus sequence type; PFGE, pulsed-field gel electrophoresis; ST, sequence type

## Additional files

Additional file 1: Bacterial species, primer sequences and PCR product size. Primers used to differentiate four bacterial species. (DOCX 34 kb)
