# Supplementary material for: Genetic and pathogenic difference between Streptococcus agalactiae serotype Ia fish and human isolates
Source: BMC Microbiol. 2016 Aug 2;16:175. doi: 10.1186/s12866-016-0794-4 (PMC4971743; doi:10.1186/s12866-016-0794-4)
Supplement: Additional file 1: — Bacterial species, primer sequences and PCR product size. Primers used to differentiate four bacterial species. (DOCX 34 kb) [file 12866_2016_794_MOESM1_ESM.docx]

Supplementary TABLE 1. Bacterial species, primer sequences and PCR product size

| Pathogen | Primers | Sequences (5’ to 3’) | Target gene | Accession No. | Product  size (bp) |
| --- | --- | --- | --- | --- | --- |
| *Lactococcus*  garvieae | V16S-f | CGGTGAAATGCGTAGAGAT | 16S rDNA | AB267905 | 586 |
|  | pLG-2 | GCACCCTCGCGGGTTG |  |  |  |
| *Streptococcus* *agalactiae* | Sdi-61 | AGGAAACCTGCCATTTGCG | ITS of 16S-23S DNA | U39765 | 192 |
|  | Sdi-252 | CAATCTATTTCTAGATCGTGG |  |  |  |
| *Streptococcus dysgalactiae* | gyrB-SDF | CCCAATTAGATGTTCGT | *gyraseB* | AB175047 | 270 |
|  | gyrB-SDR | CTCTTCTTGTTCCATAC |  |  |  |
| *Streptococcus*  *iniae* | LOX-1 | AAGGGGAAATCGCAAGTGCC | Lactate oxidase gene | Y07622 | 870 |
|  | LOX-2 | ATATCTGATTGGGCCGTCTAA |  |  |  |

All primers were designed by the combination and modification of previous primers (Chiu, 2008).
